# Immobilization of FLAG-Tagged Recombinant Adeno-Associated Virus 2 onto Tissue Engineering Scaffolds for the Improvement of Transgene Delivery in Cell Transplants

**DOI:** 10.1371/journal.pone.0129013

**Published:** 2015-06-02

**Authors:** Hua Li, Feng-Lan Zhang, Wen-Jie Shi, Xue-Jia Bai, Shu-Qin Jia, Chen-Guang Zhang, Wei Ding

**Affiliations:** 1 Department of Oral and Maxillofacial-Head and Neck Oncology, Beijing Stomatological Hospital, Capital Medical University, Beijing, China; 2 National Institutes for Food and Drug Controls, Beijing, China; 3 Beijing Institute of Genomics, Chinese Academy of Sciences, Beijing, China; 4 Department of Medical Genetics, Capital Medical University, Beijing, China; 5 Beijing Institute of Brain Disorders, Beijing, China; University of North Carolina at Chapel Hill, UNITED STATES

## Abstract

The technology of virus-based genetic modification in tissue engineering has provided the opportunity to produce more flexible and versatile biomaterials for transplantation. Localizing the transgene expression with increased efficiency is critical for tissue engineering as well as a challenge for virus-based gene delivery. In this study, we tagged the VP2 protein of type 2 adeno-associated virus (AAV) with a 3×FLAG plasmid at the N-terminus and packaged a FLAG-tagged recombinant AAV2 chimeric mutant. The mutant AAVs were immobilized onto the tissue engineering scaffolds with crosslinked anti-FLAG antibodies by N-succinimidyl-3-(2-pyridyldithiol) propionate (SPDP). Cultured cells were seeded to scaffolds to form 3D transplants, and then tested for viral transduction both *in vitro* and *in vivo*. The results showed that our FLAG-tagged AAV2 exerted similar transduction efficiency compared with the wild type AAV2 when infected cultured cells. Following immobilization onto the scaffolds of PLGA or gelatin sponge with anti-FLAG antibodies, the viral mediated transgene expression was significantly improved and more localized. Our data demonstrated that the mutation of AAV capsid targeted for antibody-based immobilization could be a practical approach for more efficient and precise transgene delivery. It was also suggested that the immobilization of AAV might have attractive potentials in applications of tissue engineering involving the targeted gene manipulation in 3D tissue cultures.

## Introduction

Adeno-associated virus (AAV) is used as a common vector for the gene therapy of a broad range of human genetic or metabolic diseases. In AAV clinical applications, the viruses are introduced through different routes of administration, including intramuscular, intravenous and intraocular administrations, where skeletal muscles, liver, ocular, heart and central nervous system are intended as the tissue delivery targets [1–5]. Dated to June 2014, 117 clinical trials using AAV as therapeutic vectors have been reported to Wiley database to cure cystic fibrosis; hemophilia B; Leber’s congenital amaurosis; neurological diseases and other disease, but 108 trials are still I or II clinical stage [6]. Concerns still exist concerning whether and how much the diffusion of injected viruses might affect the therapeutic results or such viral mobility might provoke non-desired side effects [7–9].

In recent studies in the bioengineering field, various scaffold materials have been extensively applied, some of which are involved in genetic manipulation of the implanted cells. Viral vectors, such as adenovirus or AAV were tested with lyophilized scaffolds [10, 11] and improved transgene expression with reduced immune reactions was indicated. In these reports, certain antibody crosslinking system mediated by SPDP linker for the viral immobilization onto the collagen coated scaffold surface was the most commonly used strategy, and was promisingly demonstrated to anchor adenoviruses in the scaffolds to embed into and infect heart tissues. Collagen was biocompatible and less capable of inflammatory response *in vivo*, and therefore often first considered to be used as an endogenous biological polymers[12–14]. SPDP is able to chemically fuse two types of proteins as a dual crosslinker. The crosslinking reaction using SPDP is robust and easy to operate, and can be extensively used for both laboratory or industrial purposes [15–17].

A major obstacle of AAV being used in tissue engineering with the above mentioned strategy of immobilization, is the lack of a suitable antibody able to recognize the native viral capsid and at the same time have enough specificity which is different from the natural AAV human antibodies in the serum. In the present study, we took advantage of the recent progress for the recombinant AAV technology by constructing and producing a novel functional AAV2 with FLAG tags (Hereafter referred to as ‘FL-AV2’) displayed at the viral capsid. Following this, commercially available FLAG antibody was applied to immobilize the FL-AV2 onto the tissue engineering scaffolds with collagen coated surface by SPDP linker. The application of the loaded scaffolds with immobilized AAVs were further tested for both *in vitro* and *in vivo* transgene delivery efficiencies.

## Materials and Methods

### Cell culture

The 293T, CHO, 3T3-L1 and HeLa cells (ATCC) for this study were cultured in Dulbecco modified Eagle medium, DMEM (HyClone, USA) containing 10% fetal bovine serum (Gibco, USA) at 37°C and 5% CO_2_.

### Construction and identification of FLAG-VP2 fusion plasmid

The VP2 DNA fragment was amplified by PCR from the pAAV2-RC (Stratagene, USA), the upstream primer was TCG ATA GAT CT*g ata tc*G GCT CCG GGA AAA AAG AGG, and the downstream primer was CG*g gat cc*T TAC AGA TTA CGA GTC A (the italic letters were the cloning restriction sites of *EcoR*V and *BamH*I). Using p3×FLAG-CMV-10 (Sigma Aldrich) as the backbone vector, the VP2 sequence was inserted in-frame. The resulting construct was named pFLG-VP2. The DH5α strain of *E coli* was used for transformation and plasmid amplification. The successful FLAG and VP2 fusion in the pFLG-VP2 plasmid were identified by restriction enzyme digestion as resolved by 2% agarose electrophoresis and subsequent DNA sequencing.

### Detection of FLAG-VP2 protein expression

CHO cells were transfected with pFLG-VP2 plasmid for 24 h, then fixed with 4% paraformaldehyde and incubated with primary anti-FLAG antibody and IR700 labeled fluorescence secondary antibody. The slides were post-stained with 4,6-diamidino-2-phenylindole (DAPI). Fluorescent images were acquired with a Leica SP5 confocal microscope of 63 oil objective. The 293T cells were transfected with pFLG-VP2 for 24 h, the cells were collected and lyzed with RIPA buffer, and the subjected for western blot analysis using an anti-FLAG antibody (Roche, Germany).

### Production and purification of FL-AV2 recombinant virus

The 293T cells in 100-mm plates were transfected by calcium phosphate with plasmids of pAAV2-Helper, pAAV2-RC and pAAV2-GFP (or pAAV2-Luc) (Stratagene), in addition of pFLG-VP2 at molar ratio of 1:1:1:10. After 12 h, a medium change of fresh Dulbecco modified Eagle medium (HyClone) containing 10% fetal bovine serum (Gibco) was performed [18]. After 60 h further, the cells were collected and the viral crude lysate was prepared. The method of CsCl density gradient centrifugation was used for the purification of the obtained viruses [19].In brief, the collected cells were centrifuged at 3,000 rpm for 15 min. The precipitate was underwent freeze/thaw for three times, and then sonicated at 130 w for 6×10 s following DNase I (2000U/mL in 10% deoxycholate) for 60 min, centrifugation at 4°C, 7000 rpm for 10 min was performed. The supernatant was transferred to ultracentrifugation tube with 1.25g/mL and 1.50g/mL CsCl (MERCK) gradients and the viruses were separated and collected after the overnight ultracentrifugation at 65,000 rpm in 4°C.

The yields and titer of the obtained viruses were analyzed by qPCR. For luciferase expressing viruses, Luc as the template, the Luc gene upstream and downstream primers of ATCGG TCCCG GTGTC TTCTA T and CGGCG CCATT CTATC CGCTG GA in 0.5 μL each were mixed into 10 μL 2× SYBR premix (Dalian TaKaRa), with 0.2 μL 50×ROX Reference Dye II. Sterile deionized water was used to supplement the total volume of 20 μL. The PCR reactions were carried out with a MX-3000P real-time thermocycler (Stratagene). The running parameters were set as: 1 cycle at 95°C for 10min, 40 cycles at 95°C for 30s, and 60°C for 30s, then an additional extension at 72°C for 30s. The qPCR products were further subjected to melting curve analyses for quality assurance as confirmed by 2% agarose gel electrophoresis for specificity checks. Same conditions were used for evaluations of other types of AAVs used in this study with appropriate detection primers.

### Western analyses of FLAG tagged capsid VP2 proteins

The purified FL-AV2.Luc viruses were heat-denatured and separated with 8% SDS polyacrylamide gels. Following electrophoresis, the proteins were transferred onto a nitrocellulose membrane and blocked with 10% non-fat milk in Tris-buffered saline (TBS: 50 mmol/L pH 7.5 Tris-Cl and 200 mmol/L NaCl) for 1 h. The membranes were incubated with a primary rabbit anti-FLAG antibody (1:1,000 dilutions in TBS with 1% non-fat milk and 0.5% Tween 20) at room temperature for 30 min. A secondary IR700 fluorescein-conjugated antibody from LI-COR (1:1500 dilutions) was used for additional 1 h incubation, before scanning using an Odyssey Infrared Imager (LI-COR). Similarly, a primary anti-AAV2 capsid proteins clone B1 antibody (1:1,000) and an IR Dye800CW conjugated secondary antibody (1:1,500) was used for the overlaid detection signals of the viral proteins.

### Immobilized FL-AV2 with anti-FLAG antibodies by SPDP mediated cross-linking

The cross-linking reactions was performed using 48 well tissue plates with the reaction volume of 100μL. Bovine type I collagen (Sigma-Aldrich) was used at 1mg per mL of 100 mmol/L acetic acid solution for coating the surface of the plates or tissue engineering scaffolds. The PLGA (75:25, molecular weight of 80,000 Da) was manufactured by the Institute of Shandong Medical Devices and the gelatin sponge was purchased from Jingling Pharmaceuticals, Nanjing, China. The scaffold materials were cut into approximately 8mm × 6mm × 10mm pieces before use. The cut pieces were treated with 70% alcohol for 12 h, rinsed with sterile PBS for six times, air dried, and then irradiated under UV for 30 min. The cross-linking agent 3-diaminopropyol-carbodiimide(EDAC, Sigma-Aldrich) was applied (0.1 mg/mg collagen) by incubation of 10 min at 37°C to pretreat collagen. ELISA plates were pre-coated with 10% bovine type I collagen of PBS overnight at room temperature. The reaction with 20 mmol/L SPDP (Thermo Scientific) was carried out for 4 h at room temperature [14]. After rinsed with PBS, anti-FLAG antibody (Sigma-Aldrich) of 0.5 mg/mL in 1% BSA was added for incubation at 4°C overnight. For the experimental control, a non-specific IgG mix was used instead of the FLAG antibody for the same procedures. The excess of antibodies or IgGs were removed by 6 times washed with sterile PBS, the scaffolds were air dried for storage or immediate use.

Reporter AAVs of FL-AV2.Luc, FL-AV2.GFP or FL-AV2.LacZ of 10^10^ v.g./mL dilution in 100 μL were incubated with the previous treated materials for 4 hours at room temperature, then rinsing with copious PBS and vacuum dried for storage or immediate use. A total of 5×10^4^ HeLa cells were added to each well containing the virus-loaded scaffolds for 48 h, prior to the expression of reporter genes were detected.

### Detection of viral mediated reporter gene expression

For the quality and titer assessment of FLAG-tagged AAVs, FL-AV2.Luc was used to infect HeLa cells and 3T3-L1 cells at the multiplicity of infection (MOI) from 1×10^1^ to 1×10^5^ viral genomes per cell. The relative luciferase activities were assayed by a Lumat LB 9507 luminometer (EG & G Berthold, Germany) using a Promega reporter assay kit at 24 h post infections.

For the infection of 3D cell cultures in PLGA or gelatin sponge scaffolds, FL-AV2.LacZ was used. The viral mediated LacZ transgene expression was detected using a β-galactosidase staining kit. Before the processed PLGA materials to be implanted to nude mice for *in vivo* studies, the viral infectious titer to cells was estimated to be approximately 8.8×10^5^ v.g. per 1,500 cells per cubic millimeter of the PLGA scaffold materials, as determined by qPCR. The transplanted PLGA materials together with the surrounding tissues were removed from the implanting surgical sites of the mice after 4 wks, during which no tumorigenic cell growth was observed. The obtained tissues were rinsed and subjected to subsequent histological processing of fixation, sectioning and staining. After fixation with 4% paraformaldehyde for 24 h followed by dehydration with 30% sucrose for 48 h, the sectioned slides were subject to β-galactosidase or H.E staining, which were observed under a Leica DM 5000B microscope. For other experiments using FL-AV2.GFP of qualitative evaluations, the cellular GFP expression was observed using a Leica DMIRB fluorescent microscope.

### Statistical analyses

The quantitative data were presented as mean ± standard error (SE) and tested with One-Way ANOVA. The charts were plotted with Microsoft Excel 2007. The statistical differences between the calculated means were considered significant at *P* < 0.05 and lower.

## Results

### Construction and identification of pFLG-VP2 plasmid

**Fig 1:**
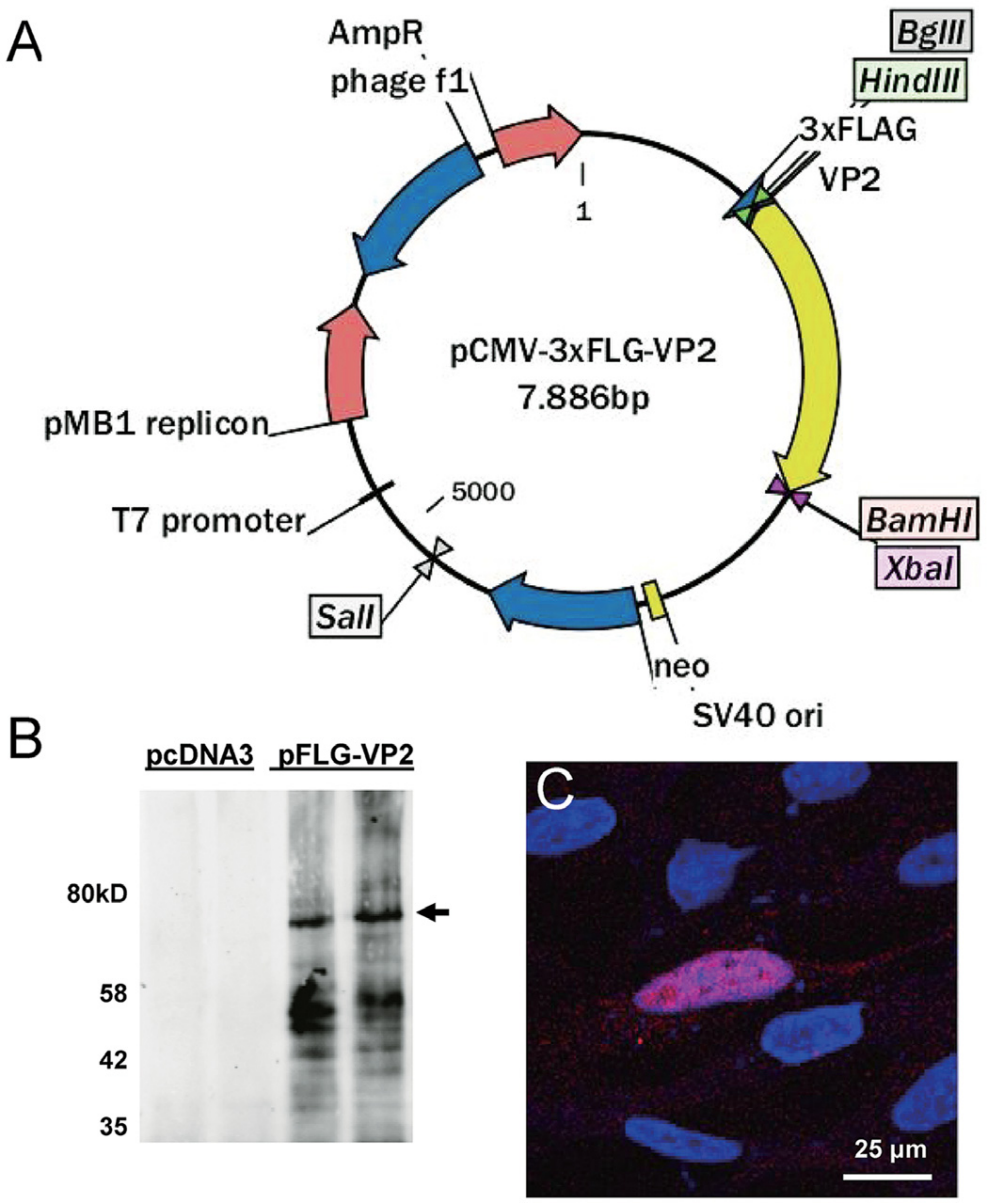
Construction of the FLAG-VP2 fusion expression plasmid. (A) The vector map of pCMV-3×FLG-VP2. (B) Identification of FLAG-VP2 expression in 293 cells by western blot analysis using primary anti-FLAG antibody and IR700 labeled fluorescence secondary antibody at 24 h post transfection. (C) Immuno-staining of FLAG-VP2 in CHO cells transfected with pCMV-3×FLG-VP2 at 48 h using an anti-FLAG polyclonal antibody.

The p3×FLAG-CMV-10 plasmid is a common and reliable vector for expressing FLAG-tagged fusion protein in eukaryotic cells. We PCR amplified the 1.8 kb VP2 coding sequence without the initial codon from the pAV2-RC plasmid, and cloned into our pCMV-3×FLAG-VP2 construct via *EcoR*V and *BamH*I double restriction digestion. This would allow efficient expression of VP2 N-terminus fusion with antigenic FLAG epitope without the possibility of false starting the translation of wild type VP2. The vector map of pFLG-VP2 was shown in Fig 1A, and the insertion was confirmed by sequencing to be fully correct. The plasmid was then amplified with DH5 as the host *E coli* strain. To test whether the FLAG-VP2 proteins could be expressed in eukaryotic cells, the AAV producer line 293T cells were transfected with the constructed plasmids. From the western blot results of the transfected cell lysate, a major dominant band at about 75 kDa of the expected size was detected using a FLAG antibody, as shown in Fig 1B.

The AAV packaging process is primarily conducted in the nuclei of the host cells, and requires the transport of VP proteins into the nuclei via the NLS (nuclear localization sequence). Despite the C-terminal NLS of VP2 in our construct being intact, we made sure our recombinant VP2 with FLAG tags was able to be transported into the nucleus by performing immunofluorescent assays to determine whether the expressed VP2 was nuclear localized. For better morphology and resolution, CHO cells were used for transfection. Indeed, the clear nuclear accumulation of FLAG-VP2 signal was observed using an anti-FLAG antibody (Fig 1C).

### Production and characterization rAAVs with FLAG-tagged VP2 in the capsids

**Fig 2:**
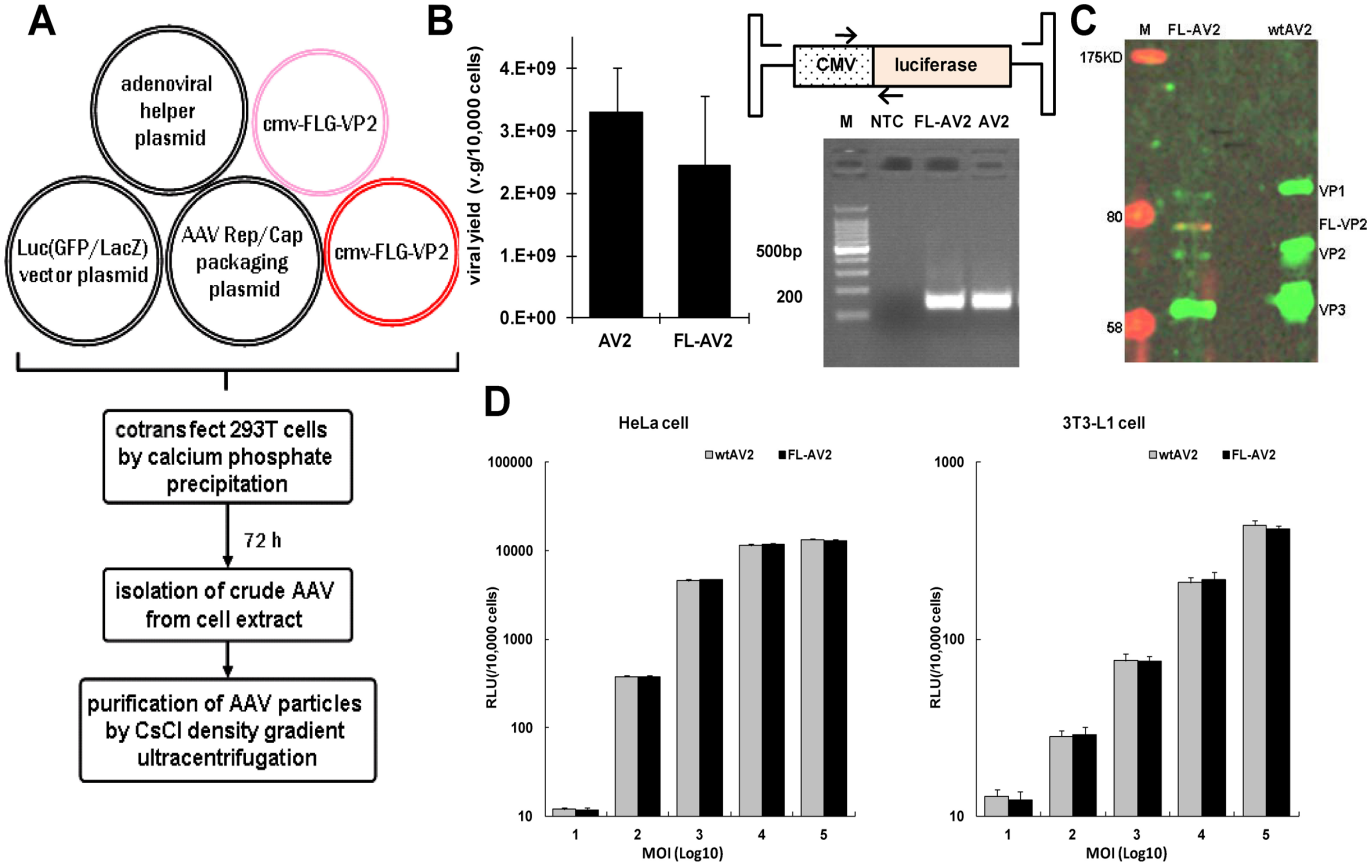
Preparation and characterization of recombinant AAV2 with FLAG-tagged capsid proteins. (A) Scheme of the AAV packaging system by cotransfection of 4 plasmids in 293T cells. (B) Comparison of the viral package yields of the 4-plasmid system (pAV-Luc:pAAV-RC:pAV-Helper:pCMV-FLG-VP2 at 1:1:1:10) with those from the conventional 3-plasmid approach (pAV-Luc:pAV-RC:pAV-Helper of equal amounts) to produce conventional viruses. The design of specific primers and their qPCR products in 2% gel electrophoresis were shown. (NTC: no template control) (C) Determination of the FLAG-VP2 incorporation in the purified FL-AV2 by western blot with the B1 anti-VP antibody (green) and anti-FLAG antibody (red). (D) Comparison of recombinant FL-AV2.Luc with regular AV2.Luc of the abilities to transduce HeLa cells (left panel) or 3T3-L1 cells (right panel). The infection doses from 1×10^1^ to 1×10^5^ virions per cell were used. The chemiluminescence measurements were carried out at 48 h after infections.

The conventional AAV packaging system uses a strategy of the co-transfection with 3 plasmids, where the viral packaged transgene, Rep/Cap protein coding genes, and helper genes from the adenovirus are constructed as individual plasmids. For our approach to produce FLAG-tagged AAVs, the FLAG-VP2 fusion plasmid was introduced as the 4^th^ plasmid for the cotransfection of the host 293T cells. To ensure the sufficient incorporation of the FLAG fusion VP2 into the viral capsid, this 4^th^ plasmid was provided in excess amount, as shown in Fig 2A. We first compared the titer yields of this approach with the conventional strategy. Taking FL-AV2.Luc for examples, the yield of purified viruses following CsCl density gradient ultracentrifugation was 2.44×10^9^ v.g./10,000 cells and the conventional 3 plasmid production of AV2.Luc was 3.31×10^9^ v.g./10,000 cells. The qPCR primers for quantitative assessment were indicated, and the PCR products were examined by agarose gel electrophoresis. The specific bands about 150 bp indicated that the analysis was reliable without contaminations (Fig 2B). From 3 independent paired production of FL-AV2.Luc and AV2-Luc, we found the packaging efficiency and viral yield were comparable, except the former appeared to be slightly less and slightly more variable.

To verify whether FLAG-VP2 was successfully packaged into the capsids of the obtained viruses, we performed SDS-PAGE of 1×10^9^ particles of the purified virus, and used both anti-FLAG (red) and anti-VP (green) antibodies for western blot. In Fig 2C, the wild type AAV2 capsids included three major proteins of VP1, VP2 and VP3 at about 1:1:10 ratio of 87, 72 and 63 kDa respectively. With our FL-VP2.Luc, an additional band at about 75 kDa between VP1 and VP2 was observed, which was also probed positive with anti-FLAG antibody. Notably, the ration of the VPs seemed to be altered in FL-AV2, probably due to the higher amount of FLG-VP2 used in our system. All the results indicated that our 4-plasmid approach was able to produce the FLAG-tagged AAV2 with the successful display the FLAG-VP2 fusions at the capsid. The amount of incorporation was about equal to the native VP2, which suggested the rate was about 50%.

We then moved forward to evaluate the viral transduction efficiency of FL-AV2 by comparing with the classic AAV2 to infect different cell line. As shown in Fig 2D, the levels of FL-AV2.Luc mediated luciferase expression was nearly the same as that of the conventional AV2.Luc in a range of titrations in both permissive (HeLa) or non-permissive (3T3-L1) cells.

### Immobilization of FL-AV2 with FLAG antibodies for the infection of 2D cell cultures

**Fig 3:**
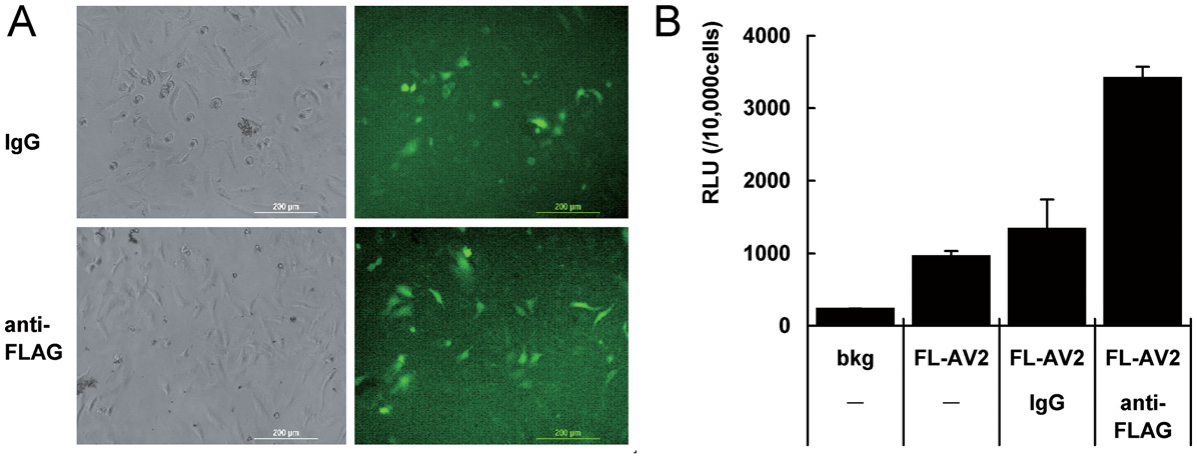
Transduction of immobilized FL-AV2 with a FLAG antibody in 2D HeLa cell cultures. ELISA plates were pre-coated with bovine type I collagen overnight. Anti-FLAG antibody or non-specific IgG mix was cross-linked collagen by incubation with using activated EDAC and SPDP. The plates were loaded with FL-AV2.GFP overnight and washed 3 times with PBS before use. HeLa cells (5×10^4^ cells/well in 48-well plates) were seeded into the plates and continuingly cultured for 48 h. Fluorescent images were acquired using a Leica DMIRB microscope. (B) Experiments under same conditions of (A) were performed using FL-AV2.Luc viruses for immobilization and infections. The quantitative chemo-luminescence was determined by luciferase assays. Measurements from 6 independent samples were averaged and presented and shown as mean+/-SE.

In order to test whether the FLAG tags in the viral capsid can be used for the immobilization of AAV2 particles, we carried out SPDP-based reactions of cross-link bovine type I collagen with anti-FLAG antibody. SPDP was a water-soluble bi-functional couplant. Type I collagen was pre-coated on the plates and activated with SPDP to introduce pyridyl-disulfide groups onto the collagen matrix. This was followed by adding the sulfhydryl-containing anti-FLAG antibody into the plates coated with the collagen pyridyl-disulfide residue, resulting in covalent attachment of the antibodies[20]. After FL-AAV2 was tethered on the processed ELISA plate, HeLa cells were seeded for 2D cultures and then analyzed for virus transduction. For easier detection of the viral mediated transgene expression, FL-AV2.GFP was produced and used for immobilization. A non-specific IgG mix was used as an experimental control. No significant change in cell attachment or growth was observed with the coating and crosslinking processes of proteins being used. Enhanced GFP expression of FL-AV2.GFP immobilized with FLAG antibody was observed compared to the samples with non-specific IgG (Fig 3A). For quantitative measures, we used FL-AV2.Luc in the immobilization system. The results showed an increase of luciferase activity, which was almost 3-fold, was detected over the FL-AV2.Luc alone or IgG crosslinking groups. The background control (bkg) was non-viral infected samples (Fig 3B). The results indicated that the FLAG tags incorporated in the AAV capsid was able to bind specific antibodies and used for viral immobilization. In addition, such specificity of antibody and engineered viral tag interactions could be used to enhance the viral transduction in 2D cell cultures.

### Immobilized FL-AV2 for the infection of 3D cell cultures in the scaffolds of PLGA and gelatin sponge

**Fig 4:**
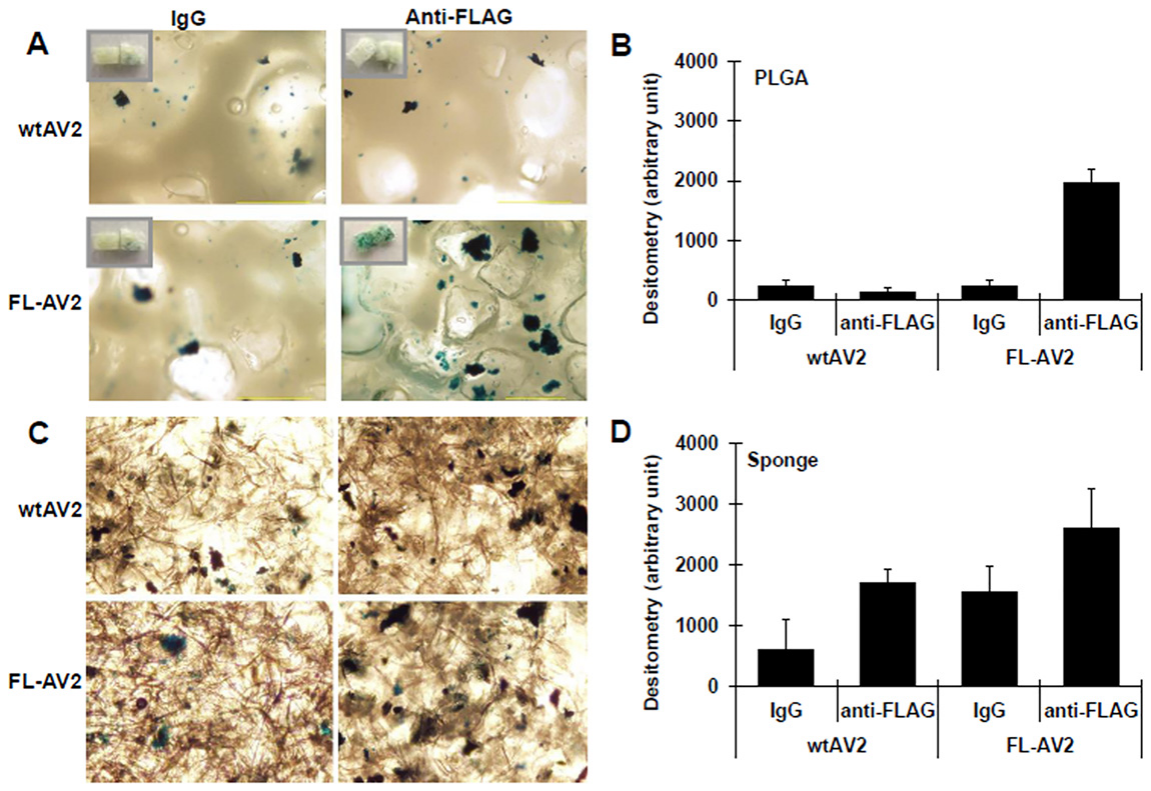
Transduction of immobilized FL-AV2 with FLAG antibodies in 3D cell cultures *in vitro*. Tissue engineering scaffolds of PLGA (A) and gelatin sponge (C) were pre-coated with bovine type I collagen. Anti-FLAG antibody or mixed IgG was cross-linked to the collagen coating. FL-AV2.LacZ was immobilized to the scaffolds overnight and clean washed with PBS. HeLa cells of 1×10^6^ cells in 1 mL were seeded and cultured for 48 h prior to ß-gal staining was performed. The 10× phase-contract images were acquired under a Leica DMIRB microscope. Randomly selected 6 non-overlapped microscopic fields were analyzed for the area percentages of β-gal stained regions(B and D), and the values were used as a semi-quantitative indication of viral transduction efficiencies.

In order to test the specificity of interactions between the antibody and engineered viral tags and whether the interaction could be used to enhance the viral transduction in 3D cell cultures, we chose the common lyophilic scaffold of PLGA and gelatin sponge for viral immobilization. PLGA and gelatin sponge were pre-coated with bovine type I collagen, and then sterilized by UV irradiation. Anti-FLAG antibody or mixed IgG was cross-linked to the collagen coating with SPDP. We have also intended to test the ordering of antibody being conjugated to collagen first, followed by collagen coating of the PLGA and gelatin in preliminary experiments, which was finally given up for usage reduction and recycling use of the expensive FLAG antibody. After wtAV2 or FL-AV2 was tethered on processed PLGA and gelatin sponge, HeLa cells were seeded for 3D cultures. The virus transduction efficiencies were analyzed by the staining of expressed transgene products. A non-specific IgG mix was used as an experimental control. For better indication of the expression of reporter gene, the wtAV2 and FL-AV2 carrying a LacZ cassette were used, which was different in the studies of 2D cultures. From the staining of whole mount materials, as well as the sections of scaffolds, enhanced β-galactosidase expression of FL-AV2.LacZ immobilized with FLAG antibody was clearly demonstrated over the cases using IgG on PLGA or gelatin sponge. However, the β-galactosidase expression of FL-AV2.LacZ with gelatin sponge was more variable than that in the PLGA scaffold, probably due to some unknown characteristic of gelatin sponge itself or its interaction with other materials used in this system (Fig 4A–4D). Therefore, in our later experiments involving the *in vivo* assessments of viral transduction efficiency, PLGA was used.

### Application of FL-AV2.LacZ immobilized with FLAG antibodies on PLGA scaffolds for *in vivo* cell transplant

**Fig 5:**
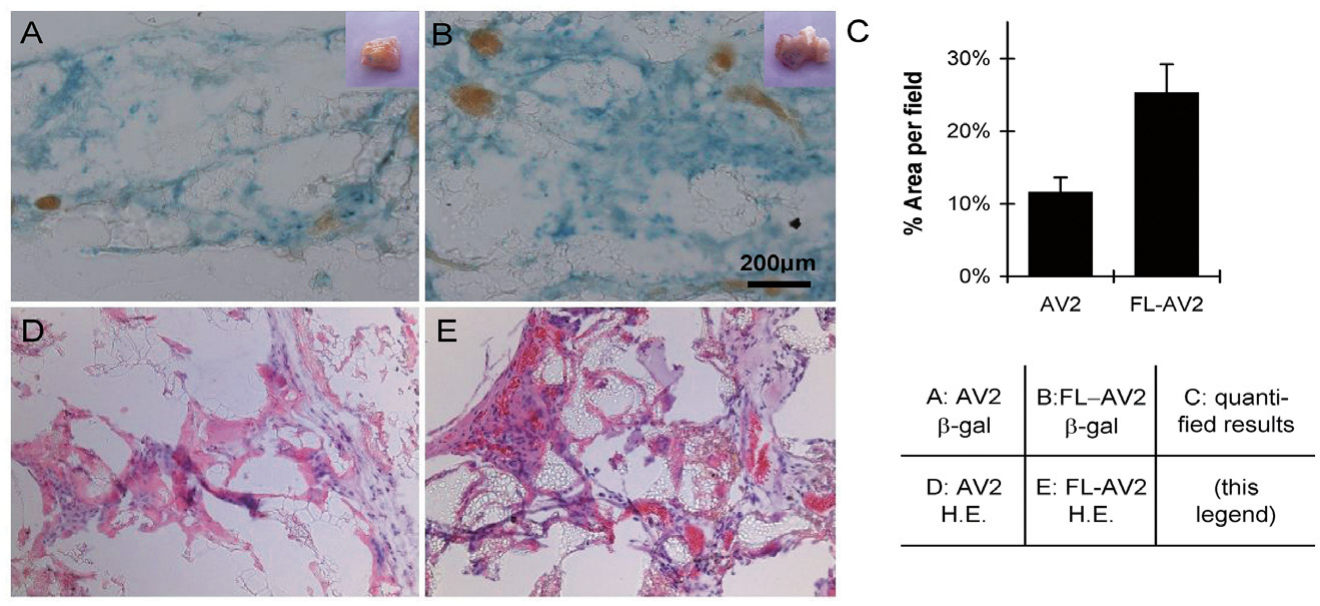
Long term transduction of antibody immobilized FL-AV2 in 3D cell cultures *in vivo*. PLGA scaffolds immobilized with FLAG antibodies were loaded with FL-AV2.LacZ and used as the 3D culture matrix for HeLa cells. After 48 h of culture, the seeded cells in scaffolds were implanted into the subcutaneous tissue under the neck skin of nude mice. After 4 weeks, the tissues surround the implanted regions were removed and subjected for histological examinations. Images of frozen sections stained with a β-galactosidase staining kit (A and B) or with H.E. staining (D and E) were obtained using a Leica DM 5000 B microscope. Randomly selected 9 non-overlapped microscopic fields were analyzed for the area percentages of β-gal stained regions(C), and the values were used as a semi-quantitative indication of viral transduction efficiencies.

To test the potential of the PLGA-based 3D culture scaffolds with immobilized AAVs for long term applications *in vivo*, implantations into nude mice were performed. The seeded HeLa cells were cultured for 48 h, and then implanted into the subcutaneous tissue under the neck skin of nude mice. A non-specific IgG mix was used as the control of the experiment. After 4 weeks, the tissues at the sites of the implantation and the surroundings were removed, clean washed, and subjected to histological examinations. As shown in Fig 5A and 5B. demonstrating the frozen section post-stained for LacZ, most of the implanted scaffolds was absorbed, and the residue PLGA could be observed as the fragmented transparent structures in the fields. From the morphological observations, the β-galactosidase expression of FL-AV2.LacZ tethered on PLGA with anti-FLAG antibody was significantly enhanced compared to the controls. From the semi-quantitative analyses of the positively stained area percentage, an increase of about two folds was detected (Fig 5A–5C). From the HE staining of the fixed tissue sections, no tumorigenic cell growth was observed. The possible explanations for this could be that HeLa cells are not the most tumorigenic lines by nature, especially when used in the form of 3D cultures and that the cell numbers we used were much less than those needed for generating *in vivo* tumor models. In the microscopic field, fibroblasts and vascular cells could be identified, but not the significant recruitment or infiltration of other cell types, like innate immune cells. (Fig 5D and 5E). Unfortunately, it was not possible differentiate in what percentage the β-gal expressing cells was originated from the implants versus the host mice cells. Presumably, the transgene positive HeLa cells would take a significant part, as implied from our *in vitro* results, together with the fact that AAV transduced HeLa cells was able to expand when implanted in nude mice.

## Discussion

### The modification of AAV2 with FLAG tag inserted VP2 protein

The advances in tissue engineering has brought various opportunities for basic biological researches and benefits in clinical applications. The combination of transgene mediated manipulation of tissues exposed in surgical or cosmetic operations is an extremely attractive area for exploration and investigation. By far, recombinant viruses were considered the most efficient vectors for transgene delivery, especially the viruses that have been trialed for gene therapies. This can be more promising for both safety and effectiveness. Among the common therapeutic viral vectors, AAV2 can be regarded as a first line choice to be used for tissue engineering purposes. This is not only because it is safe and less immunogenic, but also due to its wide spectrum of tropism for versatile tissue application. Meanwhile, the stable and robust feature of the virus itself allows harsh reactions of chemical modification without significant reduction of the viral infectivity. As the genetic engineering of AAV vectors progresses, the viable capsid protein mutations and the chimeras viral packaging technologies even expanded the utilizations of AAV as tunable gene delivery vectors, and also opened new opportunities to overcome the previous limitation for tissue engineering applications.

The immobilization of viruses to precisely transduce the targeted tissues with sufficient efficacy is a critical issue for tissue engineering applications. Specific antibodies which were not aimed for the clearance of the recombinant viruses would be prioritized and frequently used for the viral immobilization. However, in case of AAVs, A20 is current available as a sole source of AAV antibody for selective binding of intact vs. non-intact viral capsids. It can only be applied to intact AAV2 detection, the titer and specificity did not appear to be suitable for engineering applications. Besides, the cost can be intolerable even for larger scale experiments [21, 22]. The rational approach to circumvent the problem will be to modify the viral capsid and introduce proper epitopes for antibody recognition. In our study, FLAG tags were inserted into AAV2 capsid in the form of VP2 fusions.

Each AAV2 capsid is composed of roughly 60 protein subunits, including VP1, VP2 and VP3 at the ratio of 1:1:10 approximately. The C-terminus of VP3 was responsible to form the interior of AAV2 capsid structure along the folding axis, therefore it is absolutely necessary for the infectious activity and does not tolerate insertional mutations[23]. The VP1 protein contains a parvoviral encoded phospholipase A2 (pvPLA2) motif, the corresponded activity of which was suggested to be associated with an infection step following the perinuclear accumulation of infected virions, prior to and was required for the onset of early gene expression [24]. The VP2 protein was nonessential for viral infectivity, insertions in the N-terminus of VP2 fusions could be as large as a few hundreds amino acid residues such as CD34, leptin, GFP with a minimal effect on viral assembly or infectivity [23, 25]. In the present study, a pFLG-VP2 plasmid was constructed with FLAG tag inserted into N-terminus of VP2 and used with pAV-RC and a helper plasmid to produce chimeras AAV2 following a cross packaging strategy [26]. The additional pFLG-VP2 plasmid was provided in quantities that were more than traditional packaging system to ensure the sufficient incorporation of the insertion of FLAG epitopes in the viral capsids. By this approach, it limited the risk to alter VP1 proteins of the packaged virions, hence, the VP proteins naturally can be translated from a same messenger transcript. The obtained viruses were expected to maintain the nature infectivity, but were likely to lose the homogeneity of virions because of the degree of the incorporation of the fusion FLAG-VP2. However, a major concern might arise on the yield of the virus with this VP2 N-terminal fusion approach, due to the insertion of the tag, the alteration of the stoichiometry of the VPs and other factors. Indeed, insertion of tags have impact on the production of the recombinant AAVs as previously reported [23]. In our packaging system, though the stoichiometry of the VPs turned out to be altered as shown in Fig 2C, the recombinant FL-AV2 was successfully produced with a relatively satisfying yield, which was still slightly though not significantly lower than that of the wtAV2 as shown in Fig 2B. Fortunately, the FL-AV2 manifest equivalent infectious behaviors compared with wild type AV2. This has established the basis for the further investigation in its application for the transgene delivery involving FLAG antibody and tissue engineering scaffolds.

### Immobilization FLAG-tagged recombinant AAV2 to Tissue engineering scaffolds *in vitro* and *in vivo*

The advantages of AAV as a non-pathogenic, non-immunogenic and long-term expression vector [27] contributed to the safety of what administered by I.M or I.V [28–30]. However, there are concerns about virus diffusion reducing therapeutic effects or might lead to miss target side effect. Such concerns remained when AAV was used for tissue engineering to deliver genetic molecules or precursors, especially in clinical applications when lyophilization methods were preferred for locally release. In previous reports, biomaterials have been tested to directly adsorb adenovirus or AAV for in situ infections. Although the results demonstrated a successful technical route for gene transfer, damaged viral biological activities to different levels were indicated [10, 11]. This suggested that the interface of natural viral infection might be required. A remarkable study with improved results demonstrated that AAV2 was immobilized on the streptoavidin surface conjugated with Ni-NTA-biotin mediated by a hexa-histidine (6xHis) insertion into a physically-exposed loop on the capsids [31]. The hexa-histidine tag mediated vector binding to the streptoavidin surface with Ni-NTA-biotin conjugation and through optimal modulation of the balance between the level of histidine on viral surface and the level of biotin on the substrate, the virus were successfully adsorbed and released to locally delivered genes into multiple cell types. This suggested that by increasing specific recognition between viral capsid and the coating surface could be a valid strategy for optimization. However, our major concern about this hexa-histidine tag method is about its *in vivo* application, where other divalent ions in the extracellular matrix might probably interfere with the interaction of the hexa-histidine tag with the ion of Ni chelated by biotin-NTA, which would results in complication for surface immobilization.

The FLAG tag was chosen to be cloned into the AAV capsid as previously reported, since tandem FLAGs could be an more appropriate antigen for recognition and extensively used for vector engineering, including the capsid modification[32–35]. Then commercial anti-FLAG antibodies were used for the immobilization of our mutant viruses. Directly coating the scaffold surface with such antibodies is neither cost-effective nor necessary, especially for viral infections, as only small amounts of virus can be sufficient to transducer cells. Thus, we began with collagen coating, and then a typical crosslinking principle (for assays like ELISA) could be adopted. Similar experiments using the crosslinking system with SPDP linker to join adenovirus antibody for anchoring adenoviruses were attempted with satisfactory results [14, 17]. Additional interesting observations from similar reports were that the time intervals of persistent transgene expression extended [17], and the local tissue inflammation response was attenuated [36]. In our case, the better perseverance AAV infectivity was expected as it was more able to tolerate chemical and biological modifications in general. In addition, as the binding of antigens and antibodies is reversible, the FL-AAV2 activity remained normal once released from the FLAG antibody. The transgene expression was markedly improved compared to the IgG controls both *in vitro* and *in vivo*.

The 3D tissue culture system using classic or novel bioengineering materials has been rapidly developing during the past few years. Some researchers believe that such systems would resemble *in vivo* scenarios more and be better models over conventional 2D system to investigate cell behaviors. Two types of materials of PLGA and gelatin sponge were tested for FL-AAV2 immobilization and viral transduction of cells cultured in 3D forms in our study. PLGA is a polymer of lactic acid and glycolic acid, which can be degraded to carbon dioxide and water *in vivo*. PLGA in various shapes and formation has been successfully used as common scaffolds for tissue engineering for its superiority in biocompatibility and non-immunogenic property [37–39]. Gelatin sponge is a polypeptide polymer produced by partial hydrolysis of collagen protein that has been widely used as absorb stuffing in clinical and engineering scaffold [40–42]. Our finding of increased FL-AV2 mediated transgene expression as shown by β-galactosidase staining (Fig 4A–4D) was likely due to the anchoring of viruses by specific antibodies increased the local effective virus concentrations to infect the seeded cells. We also noticed that there were variations of the degrees in transduction efficiency between PLGA and gelatin sponge, which might be due to the difference in the native properties of different material used. Also, the *in vivo* results using PLGA scaffold seemed to be less promising than the *in vitro* experiments (Figs 4 and 5). Nonetheless, the successful transduction of targeted cells with the immobilization of FL-AV2 and the functional improvements over traditional AAV2 were observed to be consistent and clearly demonstrated.

### Defects and outlook

Although our study have provided important evidence suggesting the potentials of our AAV immobilization strategy to be used in 3D cultured implants of human cells for tissue engineering. The practical application of such system is worthwhile and remain to be further explored, and many questions remain to be addressed. First of all, the nude mice model of the *in vivo* studies is not quite equivalent for potential clinical applications, especially in immunological aspects. The long term effects of humoral immune responses cannot be assessed. Since the native AAV antibodies cannot be generated in such immuno-compromised mice, the exact behavior of our FLAG antibody immobilized AAV mutants, such as their interplay with immune cells, and the maintenance of the designed advantages cannot be determined at this point. However, the presented results have already outlined a possible technical route for AAV to be used in connection with current tissue engineering operations. As for today, the emerging and rapid development of three-dimensional printing technology, as well as its exciting expansion into tissue engineering fields [43, 44], the mutant virus and the immobilization principal demonstrated in this study might well be considered for certain specific applications.
